# Metagenomics and microscope revealed *T. trichiura* and other intestinal parasites in a cesspit of an Italian nineteenth century aristocratic palace

**DOI:** 10.1038/s41598-020-69497-8

**Published:** 2020-07-29

**Authors:** Daniela Chessa, Manuela Murgia, Emanuela Sias, Massimo Deligios, Vittorio Mazzarello, Maura Fiamma, Daniela Rovina, Gabriele Carenti, Giulia Ganau, Elisabetta Pintore, Mauro Fiori, Gemma L. Kay, Alessandro Ponzeletti, Piero Cappuccinelli, David J. Kelvin, John Wain, Salvatore Rubino

**Affiliations:** 1 Department of Biomedical Science, University of Sassari, V. le San Pietro 43/B, 07100 Sassari, Italy; 2 Superintendence Archaeology of Sardinia, 07100 Sassari, Italy; 3 Department of Nature and Environmental Sciences, University of Sassari, 07100 Sassari, Italy; 4 Department of Veterinary Medicine, University of Sassari, 07100 Sassari, Italy; 5 Unaffiliated, Osilo, Italy; 6 Bob Champion Research and Educational Building, University of East Anglia, Norwich Research Park, Norwich, UK; 7 The Quadram Institute, Norwich Research Park, Norwich, UK; 8 Comunica Coop, 07100 Sassari, Italy; 9 Department of Microbiology and Immunology, Dalhousie University, Halifax, NS Canada

**Keywords:** Parasite genomics, Molecular biology

## Abstract

This study evidenced the presence of parasites in a cesspit of an aristocratic palace of nineteenth century in Sardinia (Italy) by the use of classical paleoparasitological techniques coupled with next-generation sequencing. Parasite eggs identified by microscopy included helminth genera pathogenic for humans and animals: the whipworm *Trichuris* sp., the roundworm *Ascaris* sp., the flatworm *Dicrocoelium* sp. and the fish tapeworm *Diphyllobothrium* sp. In addition, 18S rRNA metabarcoding and metagenomic sequencing analysis allowed the first description in Sardinia of aDNA of the human specific *T. trichiura* species and *Ascaris* genus. Their presence is important for understanding the health conditions, hygiene habits, agricultural practices and the diet of the local inhabitants in the period under study.

## Introduction

Paleoparasitology, the study of ancient parasites recovered from archaeological sites, is a branch of paleopathology important to understand the health conditions and lifestyle of past populations^1–5^. Classical paleoparasitology consist on the rehydration and microscopic analysis of coprolites, latrine sediments, pelvic soil of skeletons or intestines of mummified bodies, followed by identification of recovered parasite eggs basing on morphometry and other characteristics (e.g. opercula, caps and surface structures)^6^. With this approach, since the first description in 1910 of *Schistosoma haematobium* eggs in a renal tissue of an Egyptian mummy^7^, helminth eggs have been identified in coprolites, latrines, mummified bodies, and archaeological contests all over the world^4,8–15^. Despite microscopy is still a method of choice for paleoparasitological studies, it allows to identify the parasites mostly to genus level, as the eggs of related species are often indistinguishable^5,16,17^. For a better taxonomic identification, immunological, hybridization and molecular techniques were developed and used in combination with classical methods^18–21^. Molecular paleoparasitological studies are mainly based on PCR amplification and Sanger sequencing of short barcoding loci as 18S rDNA, mostly using primers for specific parasite taxa^21–24^. The recent developed next generation sequencing (NGS) allows to identify multiple taxonomic groups at the same time by direct shotgun sequencing of DNA extracted from the samples (metagenomics) or by PCR-based metabarcoding of target genes^25–29^, but the application of this technique to paleoparasitology is so far limited to few studies^22,30,31^. In Europe, human intestinal parasites were described from Palaeolithic until the middle of 1900^17^, but in Sardinia (Italy) paleoparasitological analysis of cesspits or latrines were never performed, and only an archaeobotanical study reported the presence of *Trichuris* and *Ascaris* eggs from a Bronze Age well in the Central West coast of the Island^32^. Metabarcoding of rRNA 16S and 18S, and metagenomics of coprolites isolated from archaeological contests are useful also for describing the intestinal microbiome constitution of people and animals^33–36^.

In our study, we describe the finding of parasite eggs and DNA in the sediment of a cesspit of an aristocratic Palace of the nineteenth century (the Ducal Palace) in Northern Sardinia^37^, using metagenomics and rRNA gene metabarcoding to integrate conventional morphological methods. Moreover, we describe other eukaryotes and the bacterial population of the cesspit sediment.

## Results

### Conventional paleoparasitological analysis

**Figure 1:**
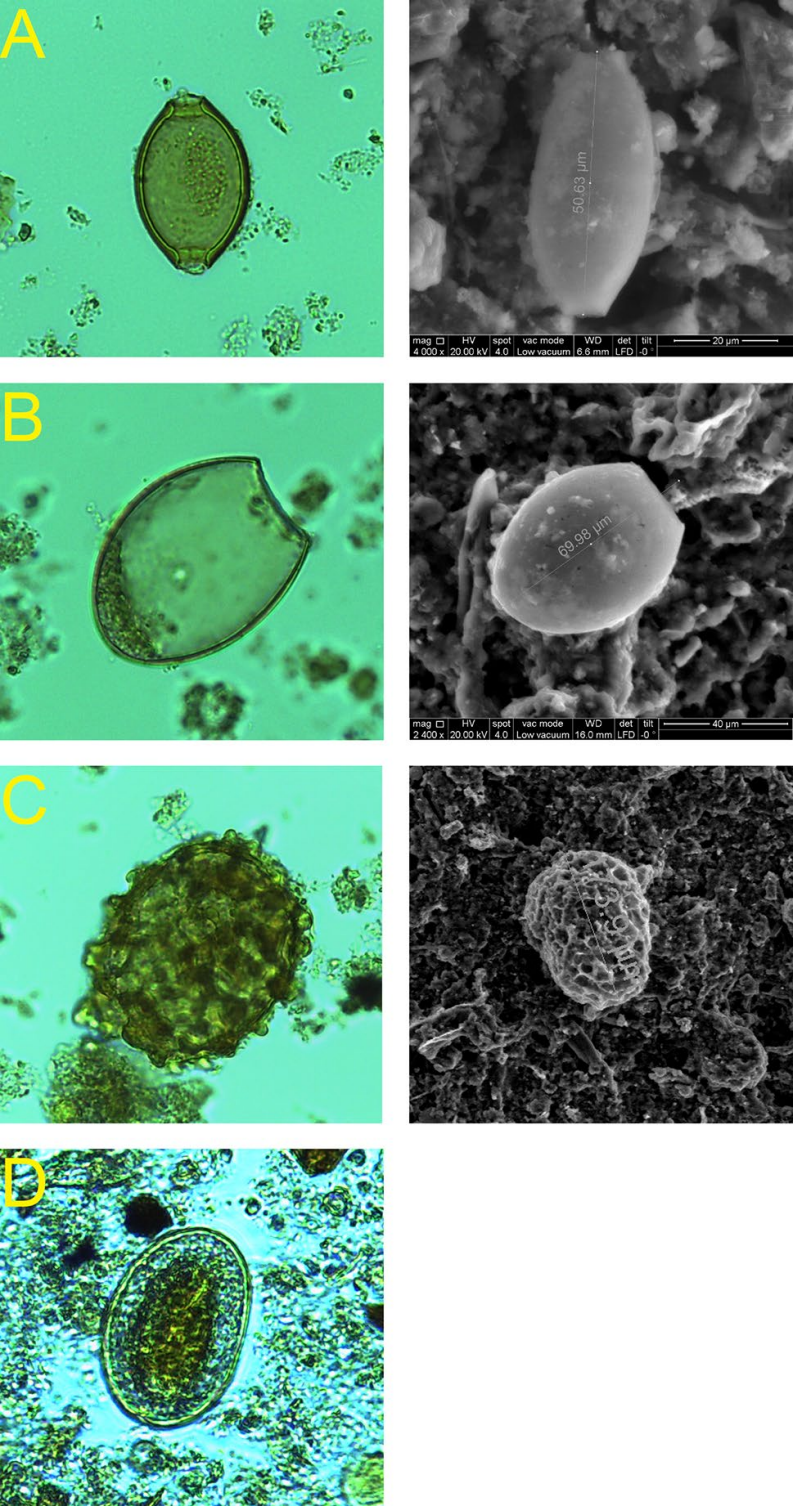
Photographs from optic and electronic microscope (left and right, respectively) of *Trichuris* sp. (**A**), *Diphyllobothrium* sp. (**B**), *Ascaris* sp. (**C**) and *Dicrocoelium* sp. (**D**).

The analysis of the cesspit sediment under light microscopy revealed the presence of diverse parasite eggs, in amber coloration (Fig. 1). We identified four different helminth taxa: the nematodes whipworm *Trichuris* sp. and roundworm *Ascaris* sp.*,* the cestode tapeworm *Diphyllobothrium* sp. and the trematode flatworm *Dicrocoelium* sp. (Fig. 1). *Trichuris* sp. appeared as the most represented genus followed by *Ascaris* sp. and *Diphyllobothrium* sp. respectively (Table 1). In the negative control sample, the absence of parasite eggs was determined after examination of fifty slides.

**Table 1:** Microscopic results of cesspit sediment from US 306. Values were obtained from observation of 150 slides.

Parasite	Eggs average number per slide	Standard deviation
*Trichuris* sp.	16	4.08
*Ascaris* sp.	10	3.02
*Diphyllobothrium* sp.	4	1.83
*Dicrocoelium* sp.	0.7	0.675

### Ancient DNA (aDNA) sequencing

#### 16S and 18S rRNA gene metabarcoding

The sequencing of the 18S rRNA amplicons generated 128,500 merged reads with an average sequence length of 126 bp and a GC content of 49.7%. Analysis identified 314 Operational Taxonomic Units (OTUs) with 33.5% of assigned reads corresponding to the phylum Nematoda, 13.9% to Chordata, 7.8% to Streptophyta, 7.8% to Amoebozoa, 6.4% to Ascomycota, 1.2% to Chlorophyta, 0.2% to Arthropoda and 0.2% to Basidiomycota (Table 2). All the reads belonging to the phylum Nematoda were assigned to the *Trichuris* genus, whereas among Amoebozoa 3% were *Acanthamoeba* and 2% *Hartmannella*.

**Table 2:** Results of 16S/18S rRNA gene and metagenomics sequencing percentage of aligned reads.

Kingdom	Phylum	Percentage of total reads from rRNA gene sequencing		Metagenomics (%)		
		16S (%)	18S (%)	US306_1	US306_2	Blank
Prokaryota	Proteobacteria	48.40		52.37	56.08	43.74
	Actinobacteria	6.70		14.99	14.91	29.45
	Firmicutes	4.70		5.66	5.11	4.96
	Planctomycetes	0.00		4.73	4.04	3.88
	Acidobacteria	14.20		4.63	4.04	3.15
	Chloroflexi	0.70		3.14	2.67	2.08
	Cyanobacteria	0.00		2.54	2.22	2.06
	Bacteroidetes	1.00		2.34	2.04	1.74
	Verrucomicrobia	0.00		1.56	1.47	1.85
	Gemmatimonadetes	9.30		1.36	1.21	1.16
	Nitrospirae	6.80		0.80	0.73	0.64
Eukaryota	Streptophyta		7.80	0.18	0.20	0.11
	Ascomycota		6.40	0.16	0.16	0.24
	Chordata		13.90	0.17	0.17	0.24
	Arthropoda		0.20	0.07	0.06	0.11
	Chlorophyta		1.20	0.06	0.06	0.02
	Basidiomycota		0.20	0.04	0.03	0.04
	Nematoda		33.50	0.03	0.03	0
	Cnidaria		0.00	0.03	0.02	0
	Amoebozoa		7.80	0	0	0
Archaea				1.37	1.37	1.37
Other		8.20	29.00	3.77	3.38	3.16
Total (%)		100	100	100	100	100

High-throughput sequencing of the 16S rRNA amplicons yielded 130,000 merged reads with an average sequence length of 126 bp and a GC content of 58%. QIIME analysis assigned about 22% of reads generating 736 OTUs comprising mainly the phyla Proteobacteria (48.4%), Acidobacteria (14.2%), Gemmatimonadetes (9.3%), Nitrospirae (6.8%), Actinobacteria (6.7%) and Firmicutes (4.7%) (Table 2)*.* Environmental bacteria were the major component at phyla level, including Gemmatimonadetes, Syntrophobacteriaceae and Rhodospirillaceae.

#### Metagenomics

The taxonomies obtained with MG-Rast for US 306-1 and US 306-2 showed that the main phyla were Proteobacteria (52% and 56%, respectively), Actinobacteria (15%) and Firmicutes (5.7% and 5.1%), whereas Eukaryota were about 1%. The main Eukaryotic phyla assigned were Streptophyta (0.18% and 0.19%), Ascomycota (0.16%), Chordata (0.17%), Arthropoda (0.07% and 0.06%), Chlorophyta (0.06%), Basidiomycota (0.04% and 0.03%), Nematoda (0.03%) and Cnidaria (0.03% and 0.02%) (Table 2). A Blank sample was also analysed on MG-Rast with the same settings obtaining 5,341 hits. Several genera were identified also in the Blank sample, even if the number of reads sequences was very low (less than 50,000). Considering the genera not identified in the Blank sample, *Ricinus* sp. (0.06% and 0.08% in US306-1 and US306-2, respectively), *Anaerococcus* sp. (0.06%), *Salmonella* sp. (0.04%), *Rattus* (0.04%) and *Homo* sp. (0.04%) were assigned among the most abundant ones in the latrine samples.

**Figure 2:**
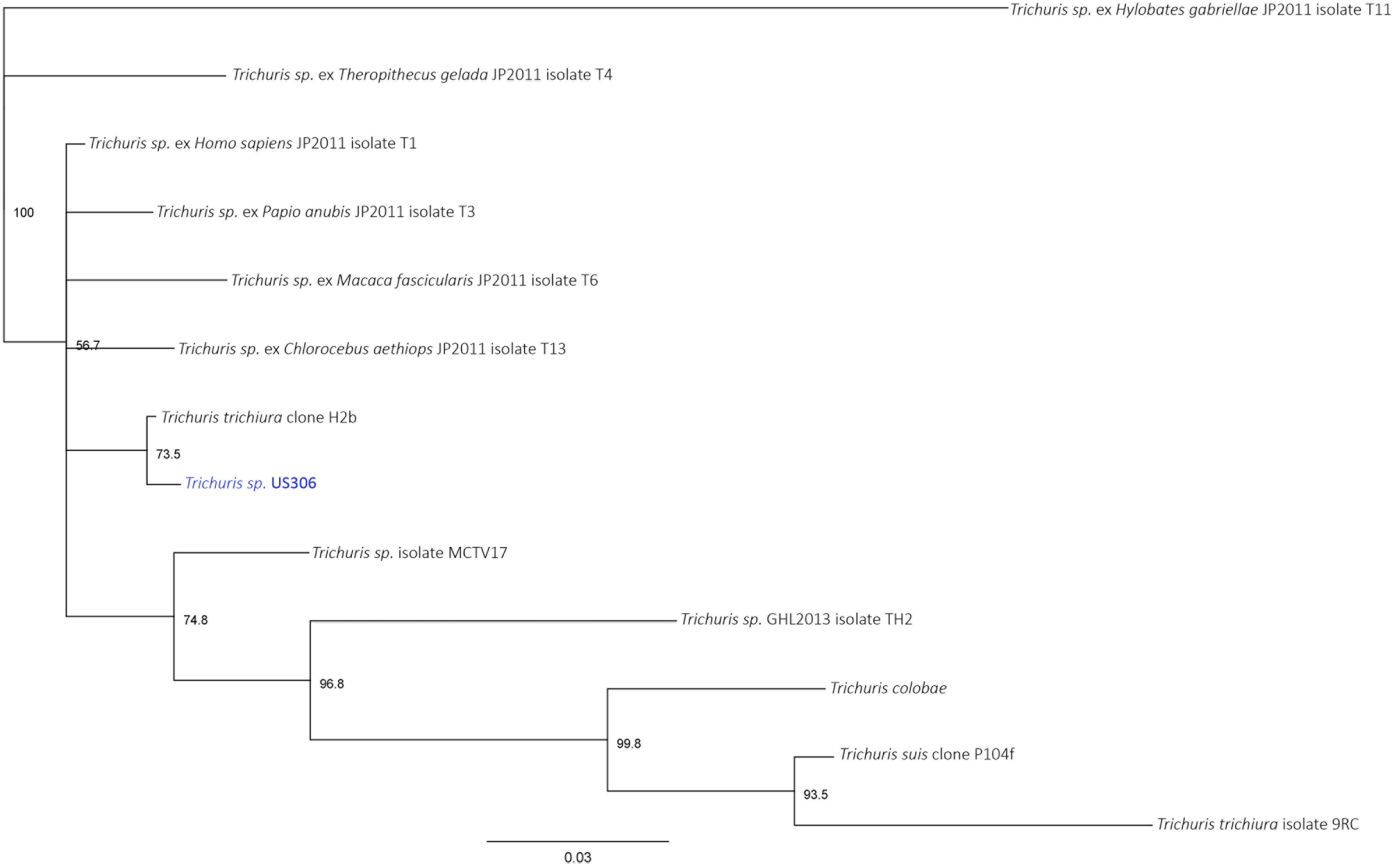
Phylogenetic tree of Trichuris sp. identified from the sample US306 (blue) and other species. In each node is indicated the consensus support and the length of the branches correspond to the number of substitutions.

**Figure 3:**
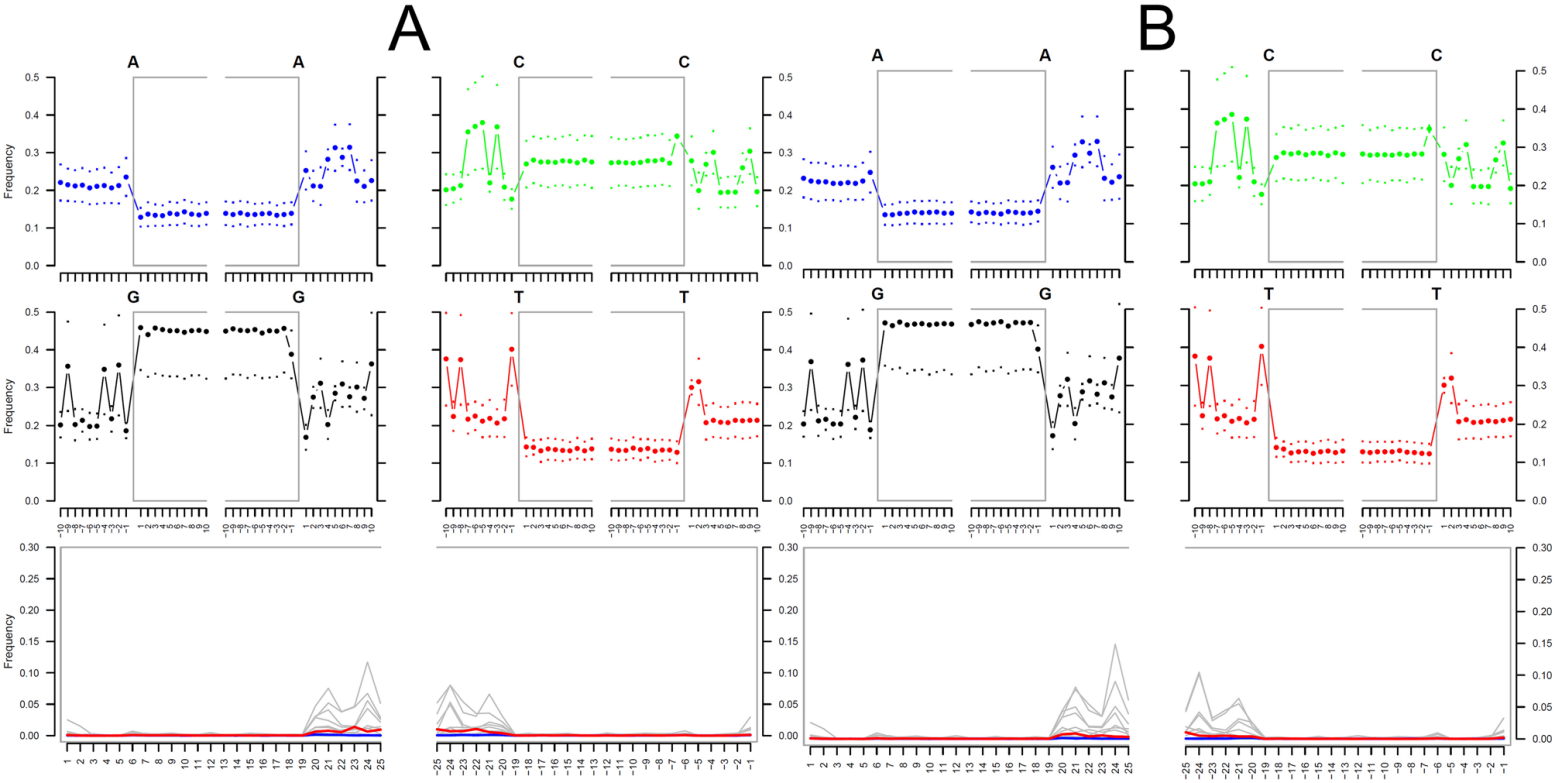
DNA damage patterns using mapDamage2 software of sequences aligned to *T. trichiura*. (**A**) US306_1; (**B**) US306_2.

Moreover, the metagenomic sequence alignments (US306-1 plus US306-2) against the genomes of the four parasites genera identified with microscopy, showed 299 reads specific for *T. trichiura* and 20 for *Ascaris* sp*.* (identity 96–100% with *A. lumbricoides* strain Ecuador, Accession LK872266). The reads specific for *T. trichiura* were used to assemble a partial ITS region long 392 bp (Supplementary Table S1). A Neighbor Joining phylogenetic tree with 1,000 bootstrap replicates was constructed with the alignment of US306 ITS and ITS sequences from different *Trichuris* sp., showing the highest similarity of our sample with *T. trichiura* clone H2b (Fig. 2). Phylogenetic trees were built with UPGMA and Maximum Likelihood methods, always showing the closeness of *Trichuris* ITS US306 with *T. trichiura* clone H2b with bootstrapping values of 74 and 88, respectively (data not shown). Reads aligned to *T. trichiura* (average distance between forward and reverse of 140 bp) were also analysed with the software mapDamage to evaluate the authenticity of ancient DNA based on the degradation of the ends of DNA sequences, exhibiting no degradation (Fig. 3).

## Discussion

Here, we report the first paleoparasitological study conducted in Sardinia using the traditional microscopy and the genomic analysis by rRNA genes metabarcoding and shotgun metagenomics in a cesspit sediment of the Ducal Palace, an aristocratic palace of Sassari that, in the first half of the nineteenth century, was inhabited by the Duke’s family and their servants. Eggs of intestinal worms are often recovered from archaeological samples because their particularly strong shells are very resistant to taphonomic processes. However, pinworm, hookworm and *Diphyllobothrium* eggs have more fragile eggshells and are mostly found in materials that have been submitted to ideal conditions of conservation, and it has been demonstrated that enclosed sites preserved parasite eggs better than open sites^12,39^. The cesspit located in the Ducal Palace offered a closed, favourable environment to the preservation of parasite eggs, in fact microscopic examination of sediment samples revealed the presence of human or animal parasites as *Trichuris* sp., *Ascaris* sp., *Diphyllobothrium* sp., and *Dicrocoelium* sp. This finding contributed to the comprehension of the health state of the inhabitants of the Palace, indicating that they were probably affected from diarrhoea and dysentery and, in case of severe infections, may have manifested malnutrition, weakness and problems in physical development^40^. Before this study, the only available information related to the health were two pharmacy jars found during the excavations containing medicine for urinary infections (named: “Antiblenorragico Valle Sassari”)^38^. To obtain a complete analysis of the microorganisms in our samples, we performed the rRNA genes metabarcoding and shotgun metagenomics on the aDNA extracted from the sediment. As the microscopy analysis of a control samples showed no presence of parasites, this sample has not been submitted to the NGS sequencing. Instead, as previous studies showed high level of contamination during sequencing^41^, metagenomics of a Blank sample was also performed. Reads assigned to several taxa like *T. trichiura*, plants and mammals were not found on Blank sample, excluding the risk of contamination. Also, the same reads showed no degradation with the software mapDamage. This result can be explained with the age of sample, around 150–200 years, not too ancient to undergo degradation of the fragments of DNA.

Regarding the parasites, 18S rRNA sequencing evidenced among the eukaryotes the prevalence of the genus *Trichuris*, which was the most abundant detected by microscopy, whereas shotgun metagenomics allowed us to identify the species *T. trichiura,* for which humans are definitive hosts, and the genus *Ascaris*. The construction of phylogenetic trees allowed us to confirm the species of *T. trichiura*, highlighting the importance of metagenomics joined to the microscope observation.

Despite the alignment of metagenomic reads to *A. lumbricoides* (specific to humans), the high genomic similarity with *A. suum* does not allow to confirm the species. On the other hand, the association of *T. trichiura* and *A. lumbricoides* is common, probably for their similar transmission cycles and ecological niches occupied, and they are the most reported human intestinal parasites from European prehistoric, medieval and historic archaeological sites^2,42^ as well as South American pre-Columbian samples^43^ and Korean soils obtained from ancient pits^13^. Particularly, *T. trichiura* eggs have been found in the famous Copper age mummy Ötzi, the iceman^44^ and *A. lumbricoides* eggs were observed in Egyptian mummies^45^. The finding of *T. trichiura* in our samples confirmed that the US 306 sediment contained human coprolites as suggested by the archaeological context^18,46,47^. Its presence is most probably linked to the huge consumption in that period, among the inhabitants of Sassari, of raw vegetables, farmed in the gardens near the houses, as descripted in some reports^48–50^, and suggests that the cesspit contents were used as fertilizer in that domestic crops. The presence of *Ascaris* in the period under study is not surprising as the stables were just in front of the Palace and the archaeozoological analysis showed animal remnants (deriving from meat consumption) and slaughter remains (the animals were slaughtered in the palace)^38^. To our knowledge, this is the first report of aDNA of *T. trichiura* and *Ascaris* sp. in Sardinia. Today, *T. trichiura* human infections are uncommon in this region, and this nematode is found mostly in rural communities of tropical and subtropical areas where is linked to malnutrition and low hygiene^17,51,52^.

In this work, *Diphyllobothrium* sp. and *Dicrocoelium* sp. were identified only by microscope, and not with NGS. This could be explained with the low number of eggs of these parasites compared to *Trichuris* and *Ascaris*, which were prevalent in our samples. Moreover, the opercula of *Dicrocoelium* eggs were open and did not preserve the DNA of the worm inside. However, differences between microscopic and genetic analyses have been already described and could be attributed to the different distribution of eggs in the samples analysed^5^.

The identification of the zoonotic pathogen *Diphyllobothrium* is very interesting as other studies have found this zoonotic pathogen in archaeological contexts related to rich and noble people^53^, similarly to our site. Its presence could be linked to the consumption of raw or undercooked fish^5^. In fact, the Duke of Modena Francis IV, in the “Description of Sardinia” reported that people in that period usually ate inadequately cooked food such as beef, chicken, fish and pork^54^. Different studies have shown that *Diphyllobothrium* was present since the Neolithic period in the Old World and from the eighth millennium before Christ (BC) on the American continent^11^. The finding of *Dicrocoelium* sp*.*, which infects mainly sheep and goats and secondarily cattle^14^, is quite interesting since sheep and bovine breeding has been practiced in Sardinia. *Dicrocoelium* eggs have been identified in Europe in a coprolite dated 550,000 years ago^55^, and during the Neolithic^2^ and the Bronze Ages^14^. *Dicrocoelium* was also identified in sediments associated with an 11th Century latrine in England ^56^. Recently in Sardinia antibodies against *Dicrocoelium dendriticum* were identified in a large number of sheep randomly chosen, indicating the diffusion of this parasite in the island^57^. However, this is the first evidence of the presence of *Dicrocoelium* and *Diphyllobotrium* in Sardinia in the past.

Other than parasites, the 18S rRNA gene sequencing and metagenomics analysis of the US 306 deposit detected the presence of DNA of Chordata and Streptophyta, among which *Rattus* sp., compatible with the several bones of rats founded in the cesspit and *Ricinus* sp., a plant very common in Sardinia^38^.

Also, was present DNA of the potentially pathogenic genera *Acanthamoeba* and *Hartmannella* that were not detected by microscopic examination. Experiments have shown that cysts once dry are very difficult to be identified, and thus paleoparasitological evidence of these protozoan parasites are rare^12,58,59^. For this reason, the use of molecular techniques may improve the identification of protozoal infections in archaeological contexts, as shown in our study.

The bacterial profiles revealed by 16S rRNA sequencing showed the prevalence of Proteobacteria, which include a wide variety of pathogens, such as *Escherichia*, *Salmonella*, *Vibrio*, *Helicobacter* and *Yersinia* and the presence of Acidobacteria that are ubiquitous, especially in soils. Interestingly, the bacterial profiles found in the nineteenth century latrine are comparable to those detected in modern public restrooms^60^.

Results obtained with metagenomics were comparable to those obtained with 16S metabarcoding, particularly with Proteobacteria being the most detected phylum with both methods. Apart for Planctomycetes, Cyanobacteria and Verrucomicrobia that were identified only with metagenomics, all the other phyla were present in both analyses. Considering most Prokaryotes in respect to Eukaryotes in the sample, results of 18S metabarcoding were also similar to metagenomics. Previous works on latrines and coprolites showed results in agreement with this analysis. For example, a study on a latrine in Belgium exhibited high percentage of Proteobacteria (58% and 85%, using metagenomics and 16S rRNA barcoding, respectively), followed by Gemmatimonadetes (15% using metagenomics)^61^. In our study we found a high percentage of Proteobacteria (48%), followed by Gemmatimonadetes (9%) using metagenomics. Other studies about ancient coprolites and mummies intestinal contents showed the presence of bacteria of *Clostridium* genus^34–36^. In our analysis, performed on a sediment (not on a pure faecal sample), *Clostridium* spp. reads exhibited a percentage of 0.51% and 0.62% in US306-1 and US306-2, respectively.

In conclusion, association of archaeological and paleo-analysis permits us to draw a picture of the lifestyle and state of health of the habitants of the Ducal palace in the middle of the nineteenth century, evidencing the diffusion of soil transmitted helminthiasis that indicate poor hygiene and proximity with animals. Combining microscopic identification with two different NGS techniques resulted in a more complete paleoparasitological identification and can be recommended as a new approach in paleoparasitological studies.

## Materials and methods

### Archaeological site

**Figure 4:**
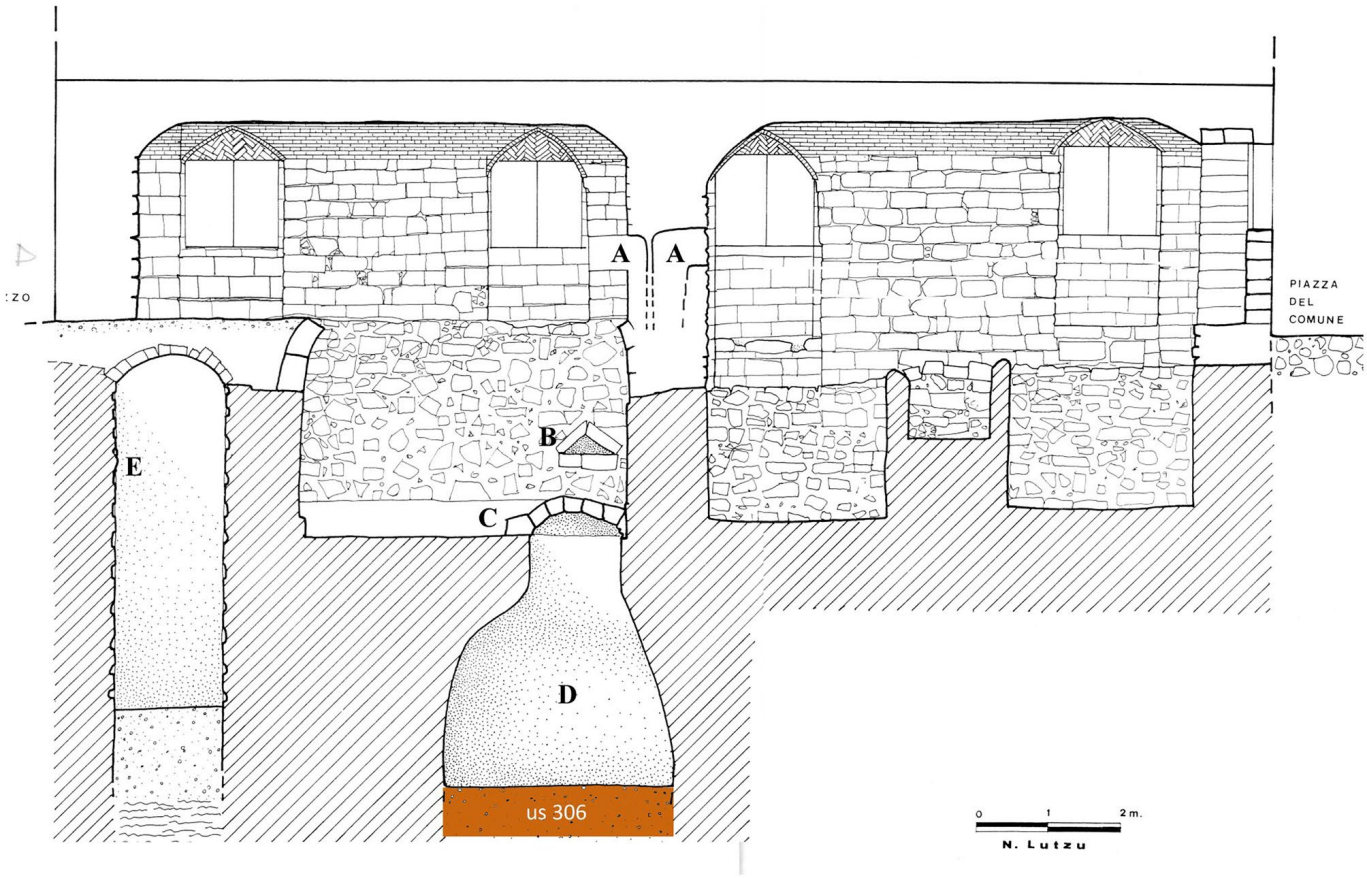
The cesspit (**D**) of the Ducal Palace. Garbage flowed into the cesspit (**D**) via two latrines (**A**) by canals carved into the rock. Domestic garbage probably was drained also from another triangle-shaped opening (**B**). The cesspit was likely emptied periodically by removing the stone slabs from the basement floor (**C**). Furthermore, a little space provided with a well is drawn in section (**E**). The cross section was adapted from the original figure granted by “Ministero per i beni e le attività culturali e per il turismo-Soprintendenza Archeologia, belle arti e paesaggio per le province di Sassari e Nuoro”.

The Ducal Palace, built between 1775 and 1804 in the city centre of Sassari (Sardinia, Italy), has been the residence of the Duke Vincenzo Manca Amat until the mid-nineteenth century. In the first decades of the 1800, the palace has been the hub of social life of the local aristocracy. From 1878, it is the seat of the Municipality of Sassari. The ground floor of the building has been subject to an archaeological intervention in 1995 during restoration work conducted by the Archaeological Superintendence of Sassari. The excavation was successively completed in 2005, bringing to light the remains of two rooms on the ground floor and five cellars interconnected and equipped with tanks, water wells and cesspools. A flask shaped cesspit excavated into the rock, about 5 m deep and with a maximum 4 m diameter, with the mouth covered with stone slabs, was located on the ground floor in the North–West corner of one cellar (Fig. 4D). The cesspit, connected to two latrines (Fig. 4A), has been reused to drain the household waste, most likely from the top of the two latrines by a triangular hole opened on the West wall (Fig. 4B), and was probably subjected to periodic emptying by removing the stone slabs from the floor pavement (Fig. 4C). At the time of the excavation, the cesspit was found partially filled with a dark brown deposit (stratigraphic unit 306, US 306) suspected to be cesspit material according to the location, the colour of sediments and the presence of presumptive coprolites. In the deposit were also found plant remains, animal bones, drinking glasses, pharmacy containers, two pistols and pottery. The analysis of the pottery, glasses and metals allowed an accurate dating of the last use of the cesspit to the first half of the nineteenth century. The US 306 deposit has been stored in sealed plastic bags and kept in the warehouse of the Archaeological Superintendent of Sassari in view of specialized studies.

### Sampling

In 2014, in order to conduct a paleoparasitological analysis, an aliquot of US 306 deposit and a negative control sampled from an area of the Palace outside the latrines were transported to the Department of Biomedical Sciences at the University of Sassari. Sampling and experiments were conducted according to the published protocols for working with aDNA^62^. Material was protected from contamination with modern DNA as follows: all procedures were performed in a controlled laboratory environment within Class II safety cabinets (Captair^®^bio, Erlab) by laboratory personnel wearing Tyvek^®^ (DuPont, USA) suits, disposable gloves and N95 masks. An aliquot of about 30 g of US 306 deposit and of the negative control were collected into sterile 50 mL Falcon tubes (DB falcon, NJ, USA) by scrapping with sterile instruments.

### Conventional paleoparasitological analysis

In order to perform parasitological observation by means of microscopy, a subsample of 1 g of US 306 deposit and another one from the negative control were suspended for 24 h in 20 ml of a 0.5% aqueous solution of trisodium phosphate, to disaggregate the soil and rehydrate the parasite remains^6^. The samples were then processed with 30% hydrochloric acid (HCl) that dissolves the calcium carbonates contained in the soil to simplify the recognition of the parasite eggs^62^. After washing out the hydrochloric acid with distilled water and centrifugation (3,000 rpm for 10 min), the samples were suspended in 5 ml of glycerol and immediately investigated under a light microscopy at magnification of 10× and 40× (Leica ICC 50 HD). In order to identify the parasite remains, 150 slides were prepared and examined. Eggs were identified according to their characteristic features and morphometry^64^.

The treatment of each sample for the ultrastructural study was performed in the following way: after the hydration of the eggs and dissolving the calcium carbonates as described before, was added 2.5% glutaraldehyde in 0.1 M Sodium cacodylate trihydrate buffer pH 7.3 and kept at rest for 24 h^63^. Subsequently the solution of each sample was pipetted and adhered on Whatman^®^ qualitative filter paper, Grade 5 (Merck KGaA, Darmstadt, Germany). After short drying, the absorbent paper surface with material has been observed in low vacuum (80 Pa) with Scanning Electron Microscope FEI Quanta 200 (Thermo Fisher Scientific).

### aDNA extraction and sequencing

All the aDNA procedures were accomplished in a controlled laboratory environment within safety cabinets and the laboratory personnel wore suits and other equipment to minimize the risk of contamination.

Total DNA was extracted from two equivalent subsamples (US 306-1 and US 306-2) of 1 g each of the original US 306 deposit using E.Z.N.A.^®^ Soil DNA Kit (Omega Bio-Tek, Georgia, USA) according to vendor’s protocol. A negative control without soil (Blank) was also processed.

#### 16S and 18S rRNA gene metabarcoding

DNA concentration and purity were estimated by Nanodrop 2000 (Thermo Scientific), then aliquots of the two samples were pooled in order to perform the sequencing. The pool DNA US 306 was amplified with universal primers for 16S rRNA (27F: AGAGTTTGATYMTGGCTCAG and 1391R: TACGGYTACCTTGTTACGACTT)^65^ and 18S rRNA (18S–82F: GAAACTGCGAATGGCTC and Ek-516R: ACCAGACTTGCCCTCC )^66,67^. Amplicons were purified and an aliquot of DNA (5 µl at 0.2 ng/µl) was sequenced at the Porto Conte Ricerche Srl (Alghero, Italy) using the Illumina HiScanSQ platform. Paired-end sequences were merged and filtered using Usearch^68^ with the following parameters: fastq_truncqual 15, fastq_minovlen 8, fastq_maxdiffs 0 and fastq_minlen 100. OTU picking was performed by QIIME 1.8.0^69^ using the GreenGenes 13_8 or Silva release 111 database (for 16S or 18S, respectively).

#### Metagenomics

DNA extracted from US 306-1 and US306-2 were quantified on a Qubit fluorometer using a dsDNA high sensitivity assay (Life Technologies, Carlsbad, California). In order to perform metagenomic sequencing, aliquots of the DNA (123 ng and 115 ng, respectively) and Blank were converted into double strand libraries using the NEBNext^®^ DNA LibraryPrep Master Mix Set for Illumina^®^ (New England BioLabs, Ipswich, MA, USA), modifying the manufacturer’s protocol as indicated by Wales et al*.*^70^. Libraries were quantified with NEBNext^®^ Library Quant Kit for Illumina (New England BioLabs, Ipswich, MA, USA), and a total of 100 µl of each library 20 µM was sent to Norwich Research Park (Norwich, UK) for sequencing with Illumina MiSeq platform. NEBNext libraries were pooled in equimolar amounts as determined by analysis on an Agilent 2200 Tapestation and HS dsDNA qubit assay (additional NexteraXT libraries were also included on the sequencing run). A total of 1.8 pM was sequenced on an Illumina NextSeq platform using the high-output v2 2 × 150 bp paired-end protocol. Metagenomic reads were merged and filtered in the same way of the rDNA sequences. After this step, the remaining sequences (581,290, 768,695 and 49,169 for US 306-1, US 306-2 and Blank, respectively) were submitted to MG-Rast^71^ with the following ID: US 306-1: mgs617078 (Accession number ERR2543096), US 306-2: mgs617081 (Accession number ERR2543095) and Blank: mgm4727577.3. The sequences were aligned against the RefSeq database with the following parameters: e-value 5, % identity 60, length 30, min. abundance 1. The pool of sequences from the two samples were also aligned against the genomes of the four parasites identified with microscopy (*T. trichiura*, Accession: CBXK000000000; *A. lumbricoides* strain Ecuador, Accession: LK872266; *D. dendriticum,* Accession: GCA_000950715; *D. latum,* Accession: GCA_000950535) using Bowtie2^72^. The specific sequences were aligned using BLAST^73^ against the NR database. Moreover, to determine the species, sequences specific of *Trichuris* sp. were aligned against the internal transcribed spacer (ITS) 2 of *T. trichiura* clone H3c (Accession: JN181845) obtaining a consensus sequence (Supplementary Table S1). That sequence was used to construct a Neighbour Joining phylogenetic tree aligning different *Trichuris* sp. using Geneious 4.8.5 (https://www.geneious.com)^74^ with the model Tamura–Nei^75^ and a bootstrap of 1,000 replicates. Also, the aligned reads to *T. trichiura* clone H3c were used to estimate the degradation using mapDamage 2.0^76^.

## Supplementary information

Supplementary information
