# Supplementary material for: Metagenomics and microscope revealed T. trichiura and other intestinal parasites in a cesspit of an Italian nineteenth century aristocratic palace
Source: Sci Rep. 2020 Jul 29;10:12656. doi: 10.1038/s41598-020-69497-8 (PMC7391740; doi:10.1038/s41598-020-69497-8)
Supplement: Supplementary file 1 — Supplementary information [file 41598_2020_69497_MOESM1_ESM.docx]

**Metagenomics and microscope revealed *T. trichiura* and other intestinal parasites in a cesspit of an Italian 19^th^ century aristocratic palace**

**Daniela Chessa^1+^, Manuela Murgia^1+*^, Emanuela Sias^1+^, Massimo Deligios^1^, Vittorio Mazzarello^1^, Maura Fiamma^1^, Daniela Rovina^2^, Gabriele Carenti^3^, Giulia Ganau^1^, Elisabetta Pintore^4^, Mauro Fiori^5^, Gemma L. Kay^6,7^, Alessandro Ponzeletti^8^, Piero Cappuccinelli^1^, David J. Kelvin^9^, John Wain^6,7^, Salvatore Rubino^1^**

^1^ Department of Biomedical Science, University of Sassari, 07100 Sassari, Italy.

^2^ Superintendence Archaeology of Sardinia, 07100 Sassari, Italy.

^3^ Department of Nature and Environmental Sciences, University of Sassari, 07100 Sassari, Italy.

^4^ Department of Veterinary Medicine, University of Sassari, 07100 Sassari, Italy.

^5^ Freelance archaeologist.

^6^ Bob Champion Research and Educational Building, University of East Anglia, Norwich Research Park, Norwich, UK.

^7^ The Quadram Institute, Norwich Research Park, Norwich, UK.

^8^ Comunica Coop., 07100 Sassari, Italy.

^9^ Department of Microbiology and Immunology, Dalhousie University, Halifax, Nova Scotia, Canada.

*Corresponding author

Manuela Murgia

Department of Biomedical Science, University of Sassari

V. le San Pietro 43/B, 07100 Sassari, Italy

Email: [manuelamurgia@hotmail.com](mailto:manuelamurgia@hotmail.com)

^+^these authors contributed equally to this work

**Supplementary Information**

| Sequence | Accession number |
| --- | --- |
| *Trichuris* sp. isolate MCTV17 | KP336475 |
| *Trichuris* sp. ex Homo sapiens JP-2011 isolate T1 | JF690940 |
| *Trichuris* sp. ex Chlorocebus aethiops JP-2011 isolate T13 | JF690949 |
| *Trichuris* sp. ex Papio hamadryas JP-2011 isolate T2 | JF690941 |
| *Trichuris trichiura* clone H2b | JN181839 |
| *Trichuris suis* clone P104f | JN181783 |
| *Trichuris colobae* | FM991956 |
| *Trichuris* sp. GHL-2013 isolate TH2 | KT186232 |
| *Trichuris trichiura* isolate 9RC | KJ588137 |
| *Trichuris* sp. ex Papio anubis JP-2011 isolate T3 | JF690942 |
| *Trichuris* sp. ex Macaca fascicularis JP-2011 isolate T6 | JF690946 |
| *Trichuris* sp. ex Hylobates gabriellae JP-2011 isolate T11 | JF690947 |
| *Trichuris* sp. ex Theropithecus gelada JP-2011 isolate T4 | JF690943 |
| *Trichuris trichiura* US306 | ERZ1195982 |
| US306-16S | ERR3680265 |
| US306-18S | ERR3680266 |
| US306_1 meta | ERR2543096 |
| US306_2 meta | ERR2543095 |
| Blank | mgm4727577.3 (Mg-Rast) |

**Supplementary Table S1.** Sequences submitted in this study.
